# Supplementary figures and images for: Measuring heterogeneity in normative models as the effective number of deviation patterns
Source: PLoS One. 2020 Nov 13;15(11):e0242320. doi: 10.1371/journal.pone.0242320 (PMC7665747; doi:10.1371/journal.pone.0242320)

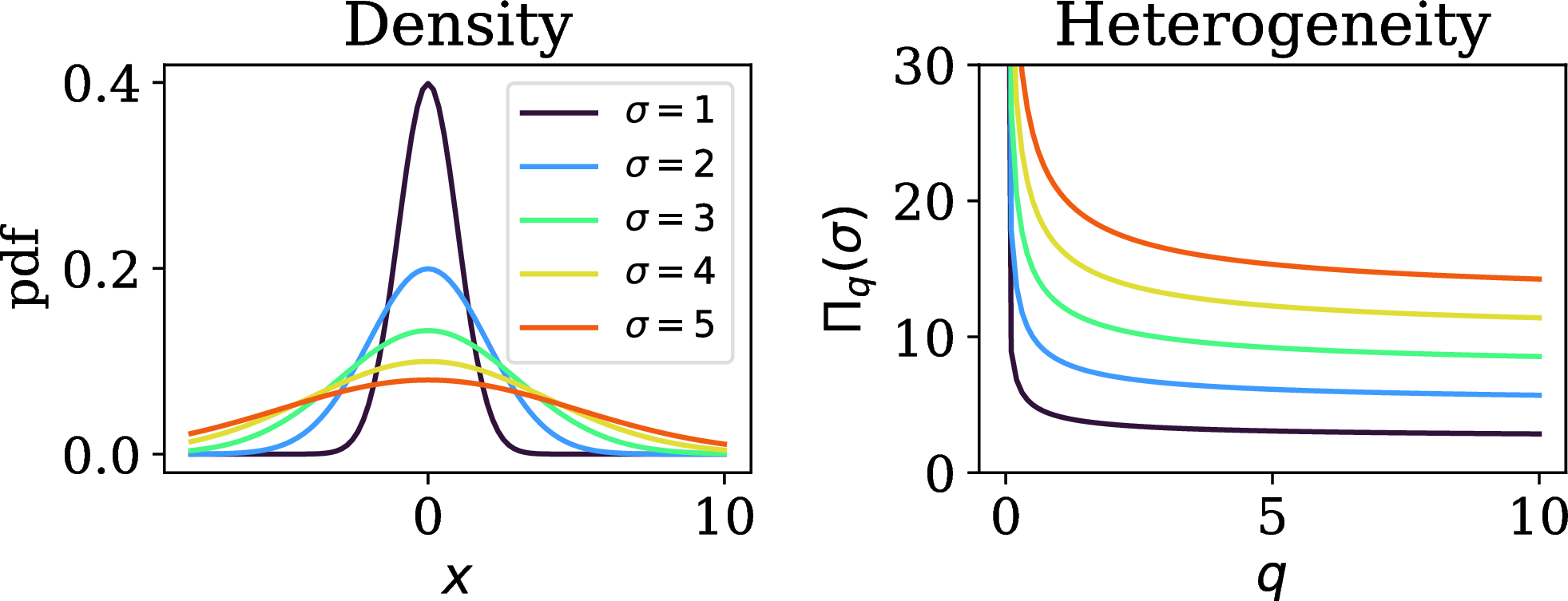

Supplement: S1 Fig — Several univariate Gaussian distributions with mean 0 and standard deviations σ1, σ2, …, σ5 (probability densities shown in the left plot), and the corresponding Rényi heterogeneity values (right plot). On a continuous domain, such as that of a Gaussian distribution, the Rényi heterogeneity has units of “effective volume” (or length, or area, depending on the dimension). (TIF) [file pone.0242320.s001.tif]

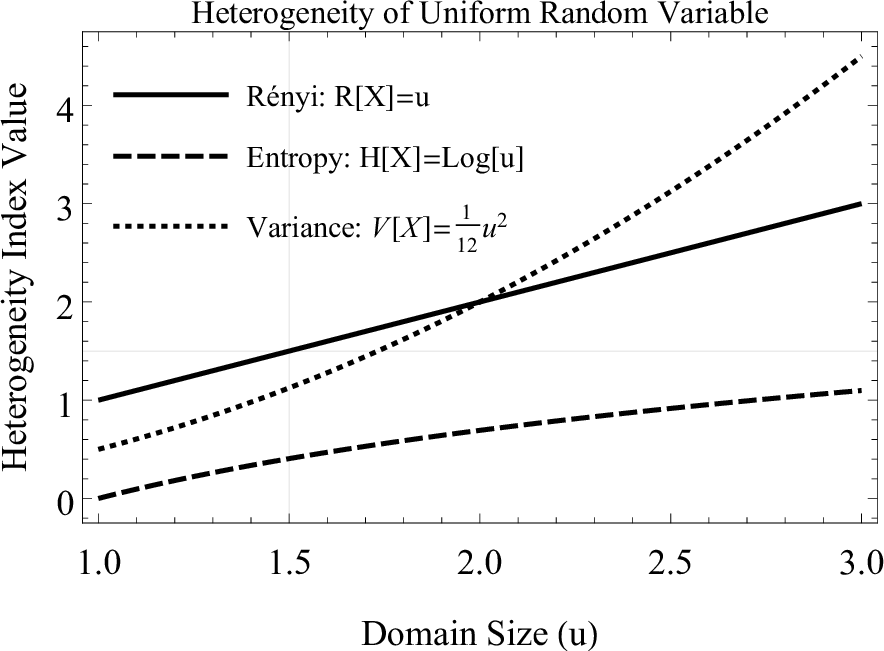

Supplement: S2 Fig — Demonstration of linear scaling of Rényi heterogeneity, in comparison to the variance and entropy on a unidimensional uniform distribution with domain size u (i.e. the domain begins at the origin). Vertical and horizontal gridlines are set at 1.5 to illustrate that the Rényi heterogeneity increases by 50% when the domain size increases by 50%. (TIF) [file pone.0242320.s002.tif]

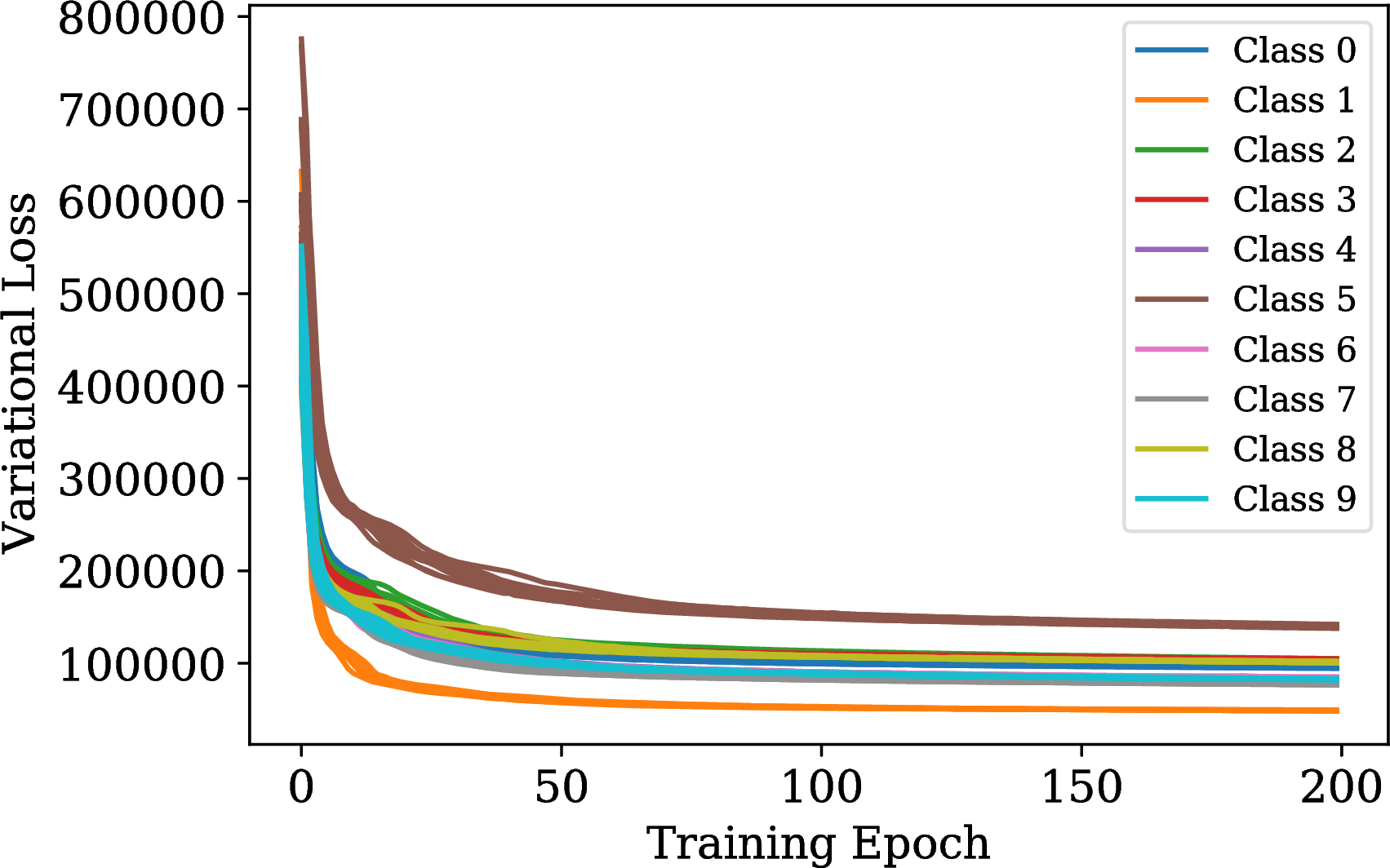

Supplement: S3 Fig — Variational loss for 10-folds of cross-validation within each digit class. (TIF) [file pone.0242320.s003.tif]

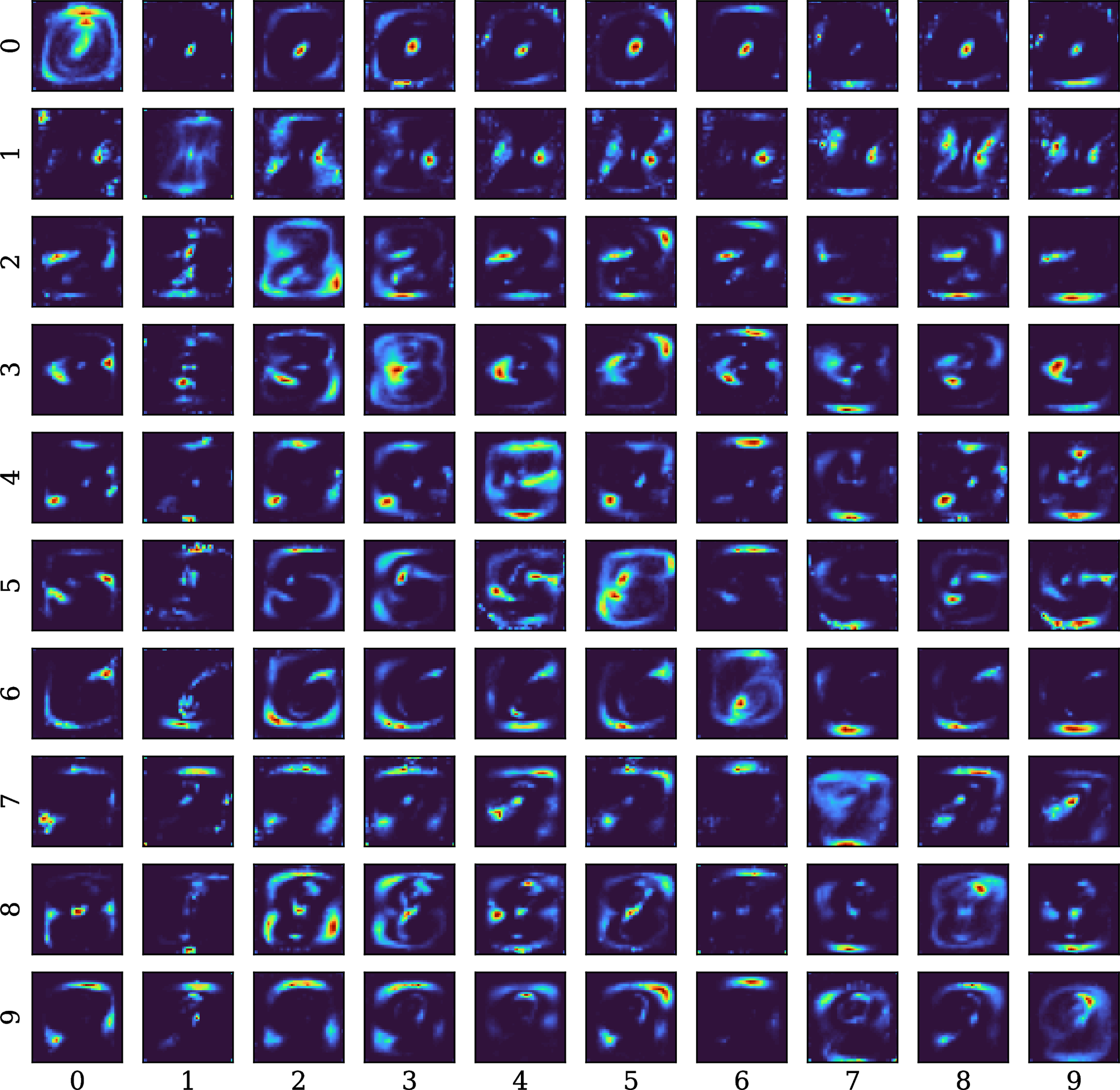

Supplement: S4 Fig — Marginal distributions of most extremely deviant pixels (at the extreme value threshold c = 0.01) for MNIST digit classes. Marginalization was done over the images of the “clinical” digit class. The digits listed along rows are the “normative” classes. The digits listed along the columns are the “clinical” cohorts. For example, the image in the top-right corner (row 0, column 9) depicts the pattern by which images of “Nines” tend to deviate from a normative distribution of “Zeros,” as modeled by a convolutional variational autoencoder [30, 31]. (TIF) [file pone.0242320.s004.tif]
