# Supplementary material for: Measuring heterogeneity in normative models as the effective number of deviation patterns
Source: PLoS One. 2020 Nov 13;15(11):e0242320. doi: 10.1371/journal.pone.0242320 (PMC7665747; doi:10.1371/journal.pone.0242320)
Supplement: Attachment — Submitted filename: ResponsesToReviewers.pdf [file pone.0242320.s012.pdf]

Abraham Nunes MD PhD MBA
Department of Psychiatry
5909 Veterans Memorial Lane
QEII Health Sciences Centre
Halifax, NS, B3H 2E2, Canada
E-Mail: nunes@dal.ca

October 9, 2020

Dear Dr. Roy,

Thank you, for considering our revised manuscript for publication in *PLOS ONE*. We have addressed reviewers' concerns in a point-by-point fashion, below. Changes in the manuscript are highlighted using the `changes` package for LaTeX.

Revisions are addressed in order of reviewers' concerns. We have quoted reviewers' concerns in gray italics, and included respective revisions in yellow-shaded box environments. We have attempted to preserve the numbering of equations as they appear in the manuscript. However, citations are numbered according to their inclusion at the end of this document, and not according to the main text.

Sincerely,

Abraham Nunes, Martin Alda, and Thomas Trappenberg

DALHOUSIE UNIVERSITY

Abraham Nunes MD PhD MBA
Department of Psychiatry
5909 Veterans Memorial Lane
QEII Health Sciences Centre
Halifax, NS, B3H 2E2, Canada
E-Mail: nunes@dal.ca

Contents

1 Responses to Academic Editor **3**
1.1 Concern 1 . . . 3
1.2 Concern 2 . . . 3
1.3 Concern 3 . . . 3

2 Responses to Reviewer 1 **5**
2.1 Concern 1 . . . 5
2.2 Concern 2 . . . 5
2.3 Concern 3 . . . 6
2.4 Concern 4 . . . 6
2.5 Concern 5 . . . 6
2.6 Concern 6 . . . 7
2.7 Concern 7 . . . 7

3 Responses to Reviewer 2 **9**
3.1 Concern 1 . . . 9
3.2 Concern 2 . . . 9

4 Responses to Reviewer 3 **10**
4.1 Concern 1 . . . 10
4.2 Concern 2 . . . 10

5 Responses to Reviewer 4 **11**
5.1 Concern 1 . . . 11
5.2 Concern 2 . . . 11
5.3 Concern 3 . . . 12
5.4 Concern 4 . . . 14
5.5 Concern 5 . . . 14
5.6 Concern 6 . . . 15
5.7 Concern 7 . . . 15

6 Tables **20**

7 Figures **21**

Abraham Nunes MD PhD MBA
Department of Psychiatry
5909 Veterans Memorial Lane
QEII Health Sciences Centre
Halifax, NS, B3H 2E2, Canada
E-Mail: nunes@dal.ca

1 Responses to Academic Editor

1.1 Concern 1

> *Please ensure that your manuscript meets PLOS ONE's style requirements, including those for file naming.*

Thank you. We believe that deviations from PLOS ONE style have been corrected. In particular, we have updated the images using the PACE Correction tool, and renamed all files.

1.2 Concern 2

> *Your ethics statement should only appear in the Methods section of your manuscript. If your ethics statement is written in any section besides the Methods, please move it to the Methods section and delete it from any other section. Please ensure that your ethics statement is included in your manuscript, as the ethics statement entered into the online submission form will not be published alongside your manuscript.*

Thank you. We have split the "Background and Methods" section into separate "Background" and "Methods" sections, such that the ethics statement is now more clearly included in the methods.

1.3 Concern 3

> *Figures captions are too long. Good to describe in text in the main manuscript.*

Thank you. We have updated the Figure 1 caption to read as follows:

> **Illustration of simulated data and normative model. Panels A-E:** Simulated distribution of five covariates. **Panel F:** Example of simulated data for $M = 30$ features and $N = 50$ subjects in a[AN] normative cohort. **Panel G:** Criticism of a normative model fit to the data in Panel A for 6 new subjects from the normative distribution (different color per subject).[AN]

We have updated the caption for Figure 2 as follows:

> **Application of normative model to independent cohorts from synthetic normative and clinical distributions. Panel A:** Simulated covariates for $N' = 100$ subjects in a clinical cohort (50 "affected" and 50 "unaffected"). **Panel B:** Plot of the $M = 30$ response variables. **Panel C:** Predictions of a normative model trained on the normative cohort data.[AN] **Panel D:** Standardized deviance (Equation 1) of each subject, at each of the 30 features.

We have updated the caption for Figure 3 as follows:

DALHOUSIE UNIVERSITY

Abraham Nunes MD PhD MBA
Department of Psychiatry
5909 Veterans Memorial Lane
QEII Health Sciences Centre
Halifax, NS, B3H 2E2, Canada
E-Mail: nunes@dal.ca

> **Measurement of heterogeneity in the synthetic clinical cohort. Panel A:** Subject-level probability distributions over the most c-deviant (i.e. top 100c%) features. Each row within a heatmap is a probability distribution computed using Equation 4, and columns show distributions across thresholds $c \in \{0.01, 0.1, 0.3, 1\}$. **Panel B:** Pooled distribution over c-deviant features ($\bar{\psi}(\mathbf{Z}^*, c)$; Equation 8), shown column-wise, across values of c (on x-axis). **Panels C-E:** The γ (Equation 7), α (Equation 9), and β heterogeneity (Equation 10), respectively, in the unaffected (blue), affected (red), and pooled (green) cohorts. Solid lines are means, and shaded ribbons are 95% confidence intervals.[AN]

The caption for Figure 4 now reads as follows:

> **Results of the MNIST experiment.** Each plot corresponds to a normative model (cVAE [1, 2]) trained on images from the "normative" digit class identified in the title. The x-axes index "clinical" or "target" digit classes.[AN] The y-axes plot the β-heterogeneity at $q = 1$ (Π_1^β, Equation 10), across thresholds $c \in \{0.01, 0.02, 0.05, 0.1\}$. Dashed vertical lines highlight maximal values of Π_1^β.[AN]

The caption for Figure 5 now reads as follows:

> **Results of experiment on ABIDE dataset. Top Row:** The effective number of deviation patterns (ENDevs; y-axes) across thresholds c for control (blue) and autism (red) subgroups. **Bottom Row:**[AN] Heterogeneity accumulation curves (ENDevs against sample size; at $c = 0.01$). The leftmost column shows results for the top $100c\%$ of absolute standardized deviance values, while centre and right columns show results for the lowest and highest $100c\%$ of standardized deviance values, respectively. Solid lines are means and ribbons are 95% confidence intervals.[AN]

DALHOUSIE UNIVERSITY

Abraham Nunes MD PhD MBA
Department of Psychiatry
5909 Veterans Memorial Lane
QEII Health Sciences Centre
Halifax, NS, B3H 2E2, Canada
E-Mail: nunes@dal.ca

2 Responses to Reviewer 1

2.1 Concern 1

The first dataset needs more information about the type of data and explanation of data like second dataset.

Thank you. We have updated the methods section to read as follows:

Experiment using Synthetic Data

Our first experiment uses synthetic data from a simple system of $M = 30$ real-valued features, $\mathbf{y}_i$—which may be considered analogous to 30 regions of interest in a neuroimaging study—using the following function parameterized by five simulated "covariates" (Fig. 1A-E):[AN]

$$\mathbf{y}_i = (x_{i1} - 2) \sin\{x_{i2}\pi\mathbf{t}\} \cos\{x_{i3}\pi\mathbf{t}\} - \cos\{\pi(\mathbf{t} - x_{i4})\} + \tilde{\boldsymbol{\epsilon}}_i, \tag{11}$$

where

$$\mathbf{t} = \left\{ \frac{(j-1)(u-l)}{M-1} + l : j \in \{1, 2, \ldots, M\}, l = -2, u = 2 \right\} \tag{12}$$

$$\tilde{\boldsymbol{\epsilon}}_i = (\tilde{\epsilon}_{ij})_{j=1,2,\ldots,M}, \;\; \tilde{\epsilon}_{ij} \sim \mathcal{N}(x_{i5}, 0.1) \tag{13}$$

and where $\mathbf{x}_i = (x_{ij})_{j=1,2,\ldots,5}$ are the covariates sampled from an isotropic multivariate Gaussian. Specific model parameters are included in the reproducible supplementary notebooks. The generative model specified by Equations 11-13 was selected in order to generate nonlinear patterns that remain easy to visualize, yet can result in non-trivial patterns of differences between simulated groups. That is, two groups simulated under these data will not simply differ along one feature dimension, but could show heterogeneous differences across multiple features.[AN]

2.2 Concern 2

Is probability distribution random?

Thank you. Yes, the probability distribution over covariates is random. We updated the subsection describing the synthetic data as follows:

We generated data from this system for $N = 50$ subjects in a "normative cohort" defined by a specific parameterization of isotropic multivariate Gaussian distribution[AN] over covariates (Fig. 1F).

DALHOUSIE UNIVERSITY

Abraham Nunes MD PhD MBA
Department of Psychiatry
5909 Veterans Memorial Lane
QEII Health Sciences Centre
Halifax, NS, B3H 2E2, Canada
E-Mail: nunes@dal.ca

2.3 Concern 3

> *"unlike variance and entropies, a 50% increase in heterogeneity corresponds to a 50% increase of variation in the system. - It needs explanation.*

Thank you, we have added a supplementary figure to illustrate this phenomenon (Figure 1 in this letter).

> In other words, unlike variance and entropies, a 50% increase in Rényi heterogeneity corresponds to a 50% increase of variation in the system (illustrated graphically in S2 Fig., but for a proof of the replication principle see Nunes, Alda, Bardouille, & Trappenberg [3])[AN].

2.4 Concern 4

> *What is c-deviant?*

Thank you. We have added the following clarifying statement to the background.

> Let $\mathbf{Z}^* = |\mathbf{Z}| = (\mathbf{z}_i^*)_{i=1,2,\dots,N}$ be the $N \times M$ absolute standardized deviance matrix, where $\mathbf{z}_i^*$ is the absolute standardized deviance for subject $i \in \{1, 2, \dots, N\}$. We set the bottom $100(1-c)\%$ of values in $\mathbf{z}_i^*$ to 0 and rescale the remainder such that they sum to one, thereby generating a probability distribution over the c-deviant features (i.e. those features with the top 100c% of standardized deviance values)[AN]

2.5 Concern 5

> *The method generated data from this system for N = 50 subjects in a "normative cohort"- How you get the value N=50 as subjects?*

Thank you. We have added the following statement to the description of the synthetic dataset.

> We generated data from this system for $N = 50$ subjects in a "normative cohort" defined by a specific parameterization of isotropic multivariate Gaussian distribution[AN] over covariates (Fig. 1F). The sample size was chosen to be large enough to allow for clear visualization of differences, while remaining small enough that the analyses could be reasonably reproduced by readers running typical personal computers. Interested readers may manipulate all parameters of this analysis in the Supplementary code (S1 File).[AN] A Gaussian process normative model was fit to these data (implemented in GPyTorch v.1.1 [4]) and evaluated for generalizability on an independently sampled normative cohort (Fig. 1G).

DALHOUSIE UNIVERSITY

Abraham Nunes MD PhD MBA
Department of Psychiatry
5909 Veterans Memorial Lane
QEII Health Sciences Centre
Halifax, NS, B3H 2E2, Canada
E-Mail: nunes@dal.ca

2.6 Concern 6

> *Sampling rate is not specified.*

Thank you. We have specified all necessary sampling rates for all analyses. In the synthetic data analysis, we generate $M = 30$ features for $N = 50$ subjects. Note that this is not a time series. In the MNIST data, there are 70,000 images across 10 classes, with the digit class-specific normative models trained using 10-fold cross-validation. In the autism dataset, there were 199 subjects in each of the control and autism groups, with the generalizability of the model evaluated under 10-fold cross-validation.

2.7 Concern 7

> *Specify result of the method in a tabular form and there is a comparative study between your method and exiting methods.*

Thank you. We have added a table to describe the results of the experiment on the ABIDE dataset (included at the end of this document as Table). We have not added the heterogeneity accumulation curves in tabular format as that table would be large and not convey the main point as well as the figures. Similarly, the results on the synthetic and MNIST data would not be better conveyed by a tabular representation of results.

Finally, there are no other approaches to measuring heterogeneity in normative modeling that can be compared to our method, so no comparative results can be offered. We have highlighted the novelty of our work in this respect in the Introduction,

> ...To date, there is no proposed method to rigorously measure heterogeneity in normative models.[AN]
> ...
>
> We therefore introduce a Rényi heterogeneity measurement for normative modeling studies, the *effective number of deviation patterns* (ENDevs), which estimates the number of distinct ways in which a cohort deviates from the normative distribution. To our knowledge, this is the first such explicitly defined heterogeneity measurement approach for the normative modeling framework.[AN] We also demonstrate a useful property of ENDevs: that cohorts sampled from *outside* a non-trivial normative distribution will tend to have lower ENDevs than cohorts sampled from the normative distribution proper.

and in the Background.

> ...Statistics summarizing deviance at the subject level can then be computed in various ways, including summation or average deviance, thresholding of the Z-score [5], or by application of extreme value statistics to model largest deviations [6]. However, there are no existing approaches for quantifying the absolute amount of heterogeneity using normative modeling.[AN]

DALHOUSIE UNIVERSITY

Abraham Nunes MD PhD MBA
Department of Psychiatry
5909 Veterans Memorial Lane
QEII Health Sciences Centre
Halifax, NS, B3H 2E2, Canada
E-Mail: nunes@dal.ca

However, we have also noted that the effective number of deviation patterns (ENDevs) is not the only possible heterogeneity measure that could be derived from normative models, and have called for further research in this respect:

> In principle, one may also derive various other RRH-derived indices for the normative modeling approach. New measures could be identified by defining other representations of deviation from the normative range; so long as one can also specify a probability distribution over such a representation, Rényi heterogeneity will be applicable, and the heterogeneity index will inherit its well understood properties [3, 7, 8].

DALHOUSIE UNIVERSITY

Abraham Nunes MD PhD MBA
Department of Psychiatry
5909 Veterans Memorial Lane
QEII Health Sciences Centre
Halifax, NS, B3H 2E2, Canada
E-Mail: nunes@dal.ca

3 Responses to Reviewer 2

3.1 Concern 1

Add motivation section.

Thank you, while we have not added a complete section on motivation, we have expanded the first paragraph of the introduction to read as follows:

> Psychiatric disorders are defined by their clinical presentations, which means that several different biological abnormalities could result in what we erroneously call a single condition. This has important consequences for biological and treatment studies that assume subjects form a single homogeneous population. Instead, in reality, a given psychiatric condition such as bipolar disorder might differ from normal population variation in heterogeneous ways. Normative modeling is a popular method for disentangling this heterogeneity in clinical cohorts[AN] [5, 9–17]. This method involves learning a distribution of normal variation, with the assumption that clinically relevant phenotypes are identifiable by significant deviations from this normative range. Unfortunately, it does not measure the *amount* of heterogeneity in a cohort. To improve our understanding of psychiatric nosology, we must aim to understand the factors that cause biological and other forms of heterogeneity in our diagnostic system, as well as the consequences of that heterogeneity on research and clinical practice. However, to study heterogeneity rigorously, we must be able to measure it rigorously. To date, there is no proposed method to rigorously measure heterogeneity in normative models.[AN]

3.2 Concern 2

Figures are also stretched.

Thank you. We believe this is an artifact of the upload to the manuscript submission server, since all images were submitted in their original sizes, which are not stretched.

Abraham Nunes MD PhD MBA
Department of Psychiatry
5909 Veterans Memorial Lane
QEII Health Sciences Centre
Halifax, NS, B3H 2E2, Canada
E-Mail: nunes@dal.ca

4 Responses to Reviewer 3

4.1 Concern 1

> *I believe the paper is well written and clearly documents the findings of the research work undertaken. I am a bit concerned about the length of the Figure captions. The Figure captions are too long.*

Thank you. We have updated the figure captions and included our changes above (Response to Academic Editor Concern 2).

4.2 Concern 2

> *Additionally, a typographic error in Line No. 178 needs to be corrected.*

Thank you. This has been corrected.

Abraham Nunes MD PhD MBA
Department of Psychiatry
5909 Veterans Memorial Lane
QEII Health Sciences Centre
Halifax, NS, B3H 2E2, Canada
E-Mail: nunes@dal.ca

5 Responses to Reviewer 4

5.1 Concern 1

The introduction section should explain the problem at hand more elaborately.

Thank you. We have updated the introduction to now read as follows.

> Psychiatric disorders are defined by their clinical presentations, which means that several different biological abnormalities could result in what we erroneously call a single condition. This has important consequences for biological and treatment studies that assume subjects form a single homogeneous population. Instead, in reality, a given psychiatric condition such as bipolar disorder might differ from normal population variation in heterogeneous ways. Normative modeling is a popular method for disentangling this heterogeneity in clinical cohorts[AN] [5, 9–17]. This method involves learning a distribution of normal variation, with the assumption that clinically relevant phenotypes are identifiable by significant deviations from this normative range. Unfortunately, it does not measure the *amount* of heterogeneity in a cohort. To improve our understanding of psychiatric nosology, we must aim to understand the factors that cause biological and other forms of heterogeneity in our diagnostic system, as well as the consequences of that heterogeneity on research and clinical practice. However, to study heterogeneity rigorously, we must be able to measure it rigorously. To date, there is no proposed method to rigorously measure heterogeneity in normative models.[AN]
>
> Heterogeneity measurement has been studied for more than a century [18], but most indices, such as variance and entropies [19–21], have inconsistent units and can scale counterintuitively [7, 22]. Conversely, ecologists and others have adopted the *Rényi heterogeneity* family of indices as a "true diversity" index [8, 23–25]. The Rényi heterogeneity measures a system's effective number of configurations (*numbers equivalent* [26]). This measure scales linearly and generalizes most commonly used diversity indices [3, 7, 8, 27, 28].
>
> We therefore introduce a Rényi heterogeneity measurement for normative modeling studies, the *effective number of deviation patterns* (ENDevs), which estimates the number of distinct ways in which a cohort deviates from the normative distribution. To our knowledge, this is the first such explicitly defined heterogeneity measurement approach for the normative modeling framework.[AN] We also demonstrate a useful property of ENDevs: that cohorts sampled from *outside* a non-trivial normative distribution will tend to have lower ENDevs than cohorts sampled from the normative distribution proper.

5.2 Concern 2

The authors have not emphasized the necessity (novelty) of this work.

Thank you, in the introduction, we have now emphasized that there are no specifically defined approaches for *measuring* heterogeneity in the normative modeling framework,

DALHOUSIE UNIVERSITY

Abraham Nunes MD PhD MBA
Department of Psychiatry
5909 Veterans Memorial Lane
QEII Health Sciences Centre
Halifax, NS, B3H 2E2, Canada
E-Mail: nunes@dal.ca

> We therefore introduce a Rényi heterogeneity measurement for normative modeling studies, the *effective number of deviation patterns* (ENDevs), which estimates the number of distinct ways in which a cohort deviates from the normative distribution. To our knowledge, this is the first such explicitly defined heterogeneity measurement approach for the normative modeling framework.[AN] We also demonstrate a useful property of ENDevs: that cohorts sampled from *outside* a non-trivial normative distribution will tend to have lower ENDevs than cohorts sampled from the normative distribution proper.

and the following added sentence highlights the importance of measuring heterogeneity in an absolute sense:

> To improve our understanding of psychiatric nosology, we must aim to understand the factors that cause biological and other forms of heterogeneity in our diagnostic system, as well as the consequences of that heterogeneity on research and clinical practice. However, to study heterogeneity rigorously, we must be able to measure it rigorously. To date, there is no proposed method to rigorously measure heterogeneity in normative models.[AN]

5.3 Concern 3

The review of past work has been covered in a sentence with a series of references; however, the readers may not be well aware of the subject. A paragraph towards the same should suffice.

Thank you. We have expanded the first paragraph of our introduction as to provide further context as follows:

> Psychiatric disorders are defined by their clinical presentations, which means that several different biological abnormalities could result in what we erroneously call a single condition. This has important consequences for biological and treatment studies that assume subjects form a single homogeneous population. Instead, in reality, a given psychiatric condition such as bipolar disorder might differ from normal population variation in heterogeneous ways. Normative modeling is a popular method for disentangling this heterogeneity in clinical cohorts[AN] [5, 9–17]. This method involves learning a distribution of normal variation, with the assumption that clinically relevant phenotypes are identifiable by significant deviations from this normative range. Unfortunately, it does not measure the *amount* of heterogeneity in a cohort. To improve our understanding of psychiatric nosology, we must aim to understand the factors that cause biological and other forms of heterogeneity in our diagnostic system, as well as the consequences of that heterogeneity on research and clinical practice. However, to study heterogeneity rigorously, we must be able to measure it rigorously. To date, there is no proposed method to rigorously measure heterogeneity in normative models.[AN]

In the Background section, we also provide a detailed description of normative modeling,

DALHOUSIE UNIVERSITY

Abraham Nunes MD PhD MBA
Department of Psychiatry
5909 Veterans Memorial Lane
QEII Health Sciences Centre
Halifax, NS, B3H 2E2, Canada
E-Mail: nunes@dal.ca

Normative Modeling

Normative modeling involves four steps [9]. First, one defines the spaces of predictor and response variables, $\mathcal{X} = \mathbb{R}^K$ and $\mathcal{Y} = \mathbb{R}^M$, respectively. A dataset $\mathcal{D}_o = \{(\mathbf{x}_i, \mathbf{y}_i) : i \in \{1, 2, \ldots, N\}\}$ is collected for a "normative" cohort comprising predictor $\mathbf{x} \in \mathcal{X}$ and response variables $\mathbf{y} \in \mathcal{Y}$. Often, the predictor variables are clinical covariates such as behavioural traits, sex, and age, with the response variable being some biological measurement such as the volume of some brain region on structural neuroimaging.

The second and third steps involve learning a model of the mapping $\mathcal{X} \to \mathcal{Y}$ using the normative cohort's data $\mathcal{D}_o$, with out-of-sample model criticism. Ideally, the model should map predictors onto a space of probability distributions on the response variable, that is $\mathbf{g} : \mathcal{X} \to \mathcal{P}(\mathcal{Y})$ [9].

Finally, one collects dataset $\mathcal{D}' = \{(\mathbf{x}'_i, \mathbf{y}'_i) : i \in \{1, 2, \ldots, N'\}\}$ from a target cohort of N' subjects, such as clinical patients. One then computes the degree to which $\mathbf{Y}'$ deviates from the predictions given by $\mathbf{g}(\mathbf{X}')$. A common approach to quantifying this deviation when $\mathbf{g}$ is a Gaussian process regression model is a Z-score that we henceforth call the "standardized deviance." For each subject $i \in \{1, 2, \ldots, N'\}$ and response variable $j \in \{1, 2, \ldots, M\}$ the standardized deviance is

$$Z_{ij} = \frac{y'_{ij} - \mu_j\left(\mathbf{x}'_i\right)}{\sqrt{\sigma_j^2\left(\mathbf{x}'_i\right) + \eta_j^2}} \tag{1}$$

where $\mu_j(\mathbf{x}'_i)$ and $\sigma_j^2\left(\mathbf{x}'_i\right)$ are the expected value and predicted variance, respectively, given predictors $\mathbf{x}'_i$, and η_j^2 is the variance in the response variable learned by the normative model. Statistics summarizing deviance at the subject level can then be computed in various ways, including summation or average deviance, thresholding of the Z-score [5], or by application of extreme value statistics to model largest deviations [6]. However, there are no existing approaches for quantifying the absolute amount of heterogeneity using normative modeling.[AN]

as well as heterogeneity measurement:

Measuring Heterogeneity

Heterogeneity is the degree to which a system deviates from perfect conformity, and is measured as the effective size of the system's event space using the Rényi heterogeneity indices [3, 24, 25, 29]. Given a system with probability distribution $\mathbf{p} = (p_i)_{i=1,2,\ldots,n}$ over n categorical states, the Rényi heterogeneity of order $q \geq 0$ is defined as follows:

$$\Pi_q(\mathbf{p}) = \begin{cases} \sum_{i=1}^n \mathbb{1}[p_i > 0] & q = 0 \\ \exp\left\{-\sum_{i=1}^n p_i \log p_i\right\} & q = 1 \\ \left(\max_i p_i\right)^{-1} & q = \infty \\ \left(\sum_{i=1}^n p_i^q\right)^{\frac{1}{1-q}} & \text{Otherwise} \end{cases}, \tag{2}$$

where $\mathbb{1}[x]$ is an indicator function. At $q \notin \{0, 1, \infty\}$, the continuous analogue for some random variable X with event space $\mathcal{X}$ and probability density function f is

DALHOUSIE UNIVERSITY

Abraham Nunes MD PhD MBA
Department of Psychiatry
5909 Veterans Memorial Lane
QEII Health Sciences Centre
Halifax, NS, B3H 2E2, Canada
E-Mail: nunes@dal.ca

$$\Pi_q(X) = \left(\int_{\mathcal{X}} f(\mathbf{x}) \mathrm{d}\mathbf{x} \right)^{\frac{1}{1-q}}, \quad (3)$$

which corresponds to the effective hypervolume of $\mathcal{X}$. The parameter q defines the indices' insensitivity to rare configurations (S1 Fig.). A critical property of Rényi heterogeneity is satisfaction of *the replication principle* [7], which guarantees that Equations 2 and 3 scale linearly with the system's number of distinct configurations [3, 28]. In other words, unlike variance and entropies, a 50% increase in Rényi heterogeneity corresponds to a 50% increase of variation in the system (illustrated graphically in S2 Fig., but for a proof of the replication principle see Nunes, Alda, Bardouille, & Trappenberg [3])[AN].

Ultimately, however, our study is the first specifically focused on how to *measure* heterogeneity using the normative modeling framework, so there is no existing comparator in this respect. We have emphasized this in the final paragraph of the introduction.

We therefore introduce a Rényi heterogeneity measurement for normative modeling studies, the *effective number of deviation patterns* (ENDevs), which estimates the number of distinct ways in which a cohort deviates from the normative distribution. To our knowledge, this is the first such explicitly defined heterogeneity measurement approach for the normative modeling framework.[AN] We also demonstrate a useful property of ENDevs: that cohorts sampled from *outside* a non-trivial normative distribution will tend to have lower ENDevs than cohorts sampled from the normative distribution proper.

5.4 Concern 4

The authors should consider explaining the images in text and reduce the lengthy explanations in figure captions.

Thank you. We have updated the figure captions and included our changes above (Response to Academic Editor Concern 2).

5.5 Concern 5

The choice of synthetic and autism dataset was understood but the use handwritten dataset was not explained elaborately (in relation to this study). Have similar studies used these datasets?

Thank you. We have updated the first paragraph of the *Experiment using the MNIST Dataset* section of the *Methods* to read as follows:

DALHOUSIE UNIVERSITY

Abraham Nunes MD PhD MBA
Department of Psychiatry
5909 Veterans Memorial Lane
QEII Health Sciences Centre
Halifax, NS, B3H 2E2, Canada
E-Mail: nunes@dal.ca

> Our first experiment used synthetic data to provide a simple illustration of how a "clinical" or "target" cohort will have a lower ENDevs than a sample of individuals drawn from the distribution of normative variation. Our second experiment seeks to evaluate whether this same phenomenon will occur with more complex and high-dimensonal data, such as natural images of objects from different categorical classes. For such a dataset to be appropriate, we must be able to guarantee that there are meaningful differences between groups in the raw data, and that these differences can be easily visualized for illustrative purposes. We therefore use the[AN] MNIST dataset, which includes 70,000 images of handwritten digits, roughly evenly balanced over classes $\{0, 1, \ldots, 9\}$ [30]. The MNIST dataset[AN] is among the simplest non-trivial examples in which consistent deviation patterns exist between robustly-defined classes. That is, (A) digit classes are valid partitions, and (B) their use for written communication mandates the existence of consistent and circumscribed deviation patterns between classes.

5.6 Concern 6

Is it possible to adapt datasets used by similar study with the proposed methodology.

Thank you. We have updated a paragraph in the discussion as follows:

> Representational Rényi heterogeneity is a general theory of heterogeneity that can be applied to any dataset, given a suitable probabilistic representation [3]. Since normative models can generate probability distributions over patterns of deviation from normal variation, RRH (here with units of ENDevs) can therefore be measured in any normative modeling study, although the normative modeling framework at present has been largely isolated to neuroimaging applications [9].[AN] Future work should extend normative modeling, and consequently[AN] our heterogeneity measure, to genetic, connectomic, and behavioural data.

5.7 Concern 7

Discussion section should include comparative study with other similar work (at least 2-3 articles that have approached the problem at hand).

Thank you. The following modification was made to the introduction to explain that there have been no previous attempts to measure heterogeneity using normative models.

> We therefore introduce a Rényi heterogeneity measurement for normative modeling studies, the *effective number of deviation patterns* (ENDevs), which estimates the number of distinct ways in which a cohort deviates from the normative distribution. To our knowledge, this is the first such explicitly defined heterogeneity measurement approach for the normative modeling framework.[AN] We also demonstrate a useful property of ENDevs: that cohorts sampled from *outside* a non-trivial normative

DALHOUSIE UNIVERSITY

Abraham Nunes MD PhD MBA
Department of Psychiatry
5909 Veterans Memorial Lane
QEII Health Sciences Centre
Halifax, NS, B3H 2E2, Canada
E-Mail: nunes@dal.ca

> distribution will tend to have lower ENDevs than cohorts sampled from the normative distribution proper.

However, to provide further clarification on the chain of papers leading to the present study, we have modified a paragraph of the discussion to read as follows:

> Our method exploits a potential synergy between normative modeling [9] and representational Rényi heterogeneity (RRH) [3]. The RRH theory was developed after previous work showed that there were no existing heterogeneity measures capable of application to the types of data used in modern psychiatric research [18, 31]. Furthermore, RRH was shown to generalize heterogeneity measures used across multiple disciplines, such as ecology [7, 24], economics [25], and statistical physics [28].[AN] Representational Rényi heterogeneity involves measuring the size of a system's event space, which can be generated (along with a probability distribution over it) by a normative model. In the present study, this was the space of c-deviation patterns, where c was an extreme value threshold. If this set of deviation patterns constitutes a relevant representation for the condition of scientific interest, then Rényi heterogeneity will provide an axiomatically sound measure of diversity in that set [7, 8, 18, 22, 29].

References

[1] Diederik P. Kingma and Max Welling. Auto-Encoding Variational Bayes. *ICLR 2014*, 2014.

[2] Diederik P. Kingma and Max Welling. An introduction to variational autoencoders. *Foundations and Trends in Machine Learning*, 12(4):307–392, 2019.

[3] A Nunes, M Alda, T Bardouille, and T Trappenberg. Representational rényi heterogeneity. *Entropy*, 22(4), 2020.

[4] Jacob R. Gardner, Geoff Pleiss, David Bindel, Kilian Q. Weinberger, and Andrew Gordon Wilson. Gpytorch: Blackbox matrix-matrix gaussian process inference with gpu acceleration, 2018.

[5] T Wolfers, CF Beckmann, M Hoogman, Jk Buitelaar, B Franke, and AF Marquand. Individual differences v. The average patient: Mapping the heterogeneity in ADHD using normative models. *Psychological Medicine*, 2019. ISSN 14698978. doi: 10.1017/S0033291719000084.

[6] Andre F. Marquand, Thomas Wolfers, Maarten Mennes, Jan Buitelaar, and Christian F. Beckmann. Beyond Lumping and Splitting: A Review of Computational Approaches for Stratifying Psychiatric Disorders. *Biological Psychiatry: Cognitive Neuroscience and Neuroimaging*, 1(5):433–447, 2016. ISSN 24519022. doi: 10.1016/j.bpsc.2016.04.002.

[7] Lou Jost. Entropy and diversity. *Oikos*, 113(2):363–375, 2006. ISSN 00301299. doi: 10.1111/j.2006.0030-1299.14714.x.

DALHOUSIE UNIVERSITY

Abraham Nunes MD PhD MBA
Department of Psychiatry
5909 Veterans Memorial Lane
QEII Health Sciences Centre
Halifax, NS, B3H 2E2, Canada
E-Mail: nunes@dal.ca

[8] Lou Jost. Partitioning Diversity into Independent Alpha and Beta Components. *Ecology*, 88(10):2427–2439, 2007. ISSN 0012-9658. doi: 10.1002/ecy.2039.

[9] Andre F. Marquand, Seyed Mostafa Kia, Mariam Zabihi, Thomas Wolfers, Jan K. Buitelaar, and Christian F. Beckmann. Conceptualizing mental disorders as deviations from normative functioning. *Molecular Psychiatry*, 2019. ISSN 14765578. doi: 10.1038/s41380-019-0441-1. URL http://dx.doi.org/10.1038/s41380-019-0441-1.

[10] Richard A.I. Bethlehem, Jakob Seidlitz, Rafael Romero-Garcia, and Michael V. Lombardo. Using normative age modelling to isolate subsets of individuals with autism expressing highly age-atypical cortical thickness features. *bioRxiv*, page 252593, 2018. doi: 10.1101/252593. URL https://www.biorxiv.org/content/early/2018/01/23/252593.1.

[11] Mariam Zabihi, Marianne Oldehinkel, Thomas Wolfers, Vincent Frouin, David Goyard, Eva Loth, Tony Charman, Julian Tillmann, Tobias Banaschewski, Guillaume Dumas, Rosemary Holt, Simon Baron-Cohen, Sarah Durston, Sven Bölte, Declan Murphy, Christine Ecker, Jan K. Buitelaar, Christian F. Beckmann, and Andre F. Marquand. Dissecting the Heterogeneous Cortical Anatomy of Autism Spectrum Disorder Using Normative Models. *Biological Psychiatry: Cognitive Neuroscience and Neuroimaging*, 4(6):567–578, 2019. ISSN 24519030. doi: 10.1016/j.bpsc.2018.11.013. URL https://doi.org/10.1016/j.bpsc.2018.11.013.

[12] Andre F Marquand, Iead Rezek, Jan Buitelaar, and Christian F Beckmann. Understanding Heterogeneity in Clinical Cohorts Using Normative Models: Beyond Case-Control Studies. *Biological Psychiatry*, 80(7):552–561, 2016. ISSN 0006-3223. doi: 10.1016/j.biopsych.2015.12.023. URL http://dx.doi.org/10.1016/j.biopsych.2015.12.023.

[13] D Kessler, M Angstadt, and C Sripada. Growth charting of brain connectivity networks and the identification of attention impairment in youth. *JAMA Psychiatry*, 73(5):481–489, 2016. ISSN 2168622X. doi: 10.1001/jamapsychiatry.2016.0088.

[14] Thomas Wolfers, Nhat Trung Doan, Tobias Kaufmann, Dag Alnæs, Torgeir Moberget, Ingrid Agartz, Jan K. Buitelaar, Torill Ueland, Ingrid Melle, Barbara Franke, Ole A. Andreassen, Christian F. Beckmann, Lars T. Westlye, and Andre F. Marquand. Mapping the Heterogeneous Phenotype of Schizophrenia and Bipolar Disorder Using Normative Models. *JAMA Psychiatry*, 75(11):1146–1155, 2018. ISSN 2168622X. doi: 10.1001/jamapsychiatry.2018.2467.

[15] Aaron F. Alexander-Bloch, Philip T. Reiss, Judith Rapoport, Harry McAdams, Jay N. Giedd, Ed T. Bullmore, and Nitin Gogtay. Abnormal cortical growth in schizophrenia targets normative modules of synchronized development. *Biological Psychiatry*, 76(6):438–446, 2014. ISSN 18732402. doi: 10.1016/j.biopsych.2014.02.010. URL http://dx.doi.org/10.1016/j.biopsych.2014.02.010.

[16] G. Ziegler, G. R. Ridgway, R. Dahnke, and C. Gaser. Individualized Gaussian process-based prediction and detection of local and global gray matter abnormalities in elderly subjects. *NeuroImage*, 97:333–348, 2014. ISSN 10959572. doi: 10.1016/j.neuroimage.2014.04.018. URL http://dx.doi.org/10.1016/j.neuroimage.2014.04.018.

DALHOUSIE UNIVERSITY

Abraham Nunes MD PhD MBA
Department of Psychiatry
5909 Veterans Memorial Lane
QEII Health Sciences Centre
Halifax, NS, B3H 2E2, Canada
E-Mail: nunes@dal.ca

[17] W. Huizinga, D. H.J. Poot, M. W. Vernooij, G. V. Roshchupkin, E. E. Bron, M. A. Ikram, D. Rueckert, W. J. Niessen, and S. Klein. A spatio-temporal reference model of the aging brain. *NeuroImage*, 169 (December 2017):11–22, 2018. ISSN 10959572. doi: 10.1016/j.neuroimage.2017.10.040.

[18] Abraham Nunes, Thomas Trappenberg, and Martin Alda. The definition and measurement of heterogeneity. *Translational Psychiatry*, 10:299, 2020. doi: 10.1038/s41398-020-00986-0. URL https://www.nature.com/articles/s41398-020-00986-0.

[19] C Tsallis. Possible generalization of Boltzmann-Gibbs statistics. *Journal of Statistical Physics*, 52(1-2): 479–487, 1988. ISSN 00224715. doi: 10.1007/BF01016429.

[20] C Gini. *Variabilità e mutabilità. Contributo allo Studio delle Distribuzioni e delle Relazioni Statistiche.* C. Cuppini, Bologna, Italy, 1912.

[21] CE Shannon. A mathematical theory of communication. *The Bell System Technical Journal*, 27 (July 1928):379–423, 1948. ISSN 07246811. doi: 10.1145/584091.584093. URL http://cm.bell-labs.com/cm/ms/what/shannonday/shannon1948.pdf.

[22] L Jost. Mismeasuring biological diversity: Response to Hoffmann and Hoffmann (2008). *Ecological Economics*, 68(4):925–928, 2009. ISSN 00319317. doi: 10.1034/j.1399-3054.2002.1150317.x. URL http://linkinghub.elsevier.com/retrieve/pii/S092180090800476X.

[23] A Rényi. On measures of entropy and information. *Proceedings of the Fourth Berkeley Symposium on Mathematical Statistics and Probability*, 1:547–561, 1963.

[24] M. O. Hill. Diversity and Evenness: A Unifying Notation and Its Consequences. *Ecology*, 54(2):427–432, 1973. ISSN 00129658. doi: 10.2307/1934352. URL http://doi.wiley.com/10.2307/1934352.

[25] Leslie Hannah and JA Kay. *Concentration in Modern Industry: Theory, Measurement, and the U.K. Experience.* The MacMillan Press, London, UK, 1977.

[26] GP Patil and C Taillie. Diversity as a Concept and its Measurement. *Journal of the American Statistical Association*, 77(379):548–561, 1982.

[27] Aisling Daly, Jan Baetens, and Bernard De Baets. Ecological Diversity: Measuring the Unmeasurable. *Mathematics*, 6(7):119, 2018. ISSN 2227-7390. doi: 10.3390/math6070119. URL http://www.mdpi.com/2227-7390/6/7/119.

[28] Iddo I. Eliazar and Igor M. Sokolov. Measuring statistical evenness: A panoramic overview. *Physica A: Statistical Mechanics and its Applications*, 391(4):1323–1353, 2012. ISSN 03784371. doi: 10.1016/j.physa.2011.09.007. URL http://dx.doi.org/10.1016/j.physa.2011.09.007.

[29] Abraham Nunes, Thomas Trappenberg, and Martin Alda. We need an operational framework for heterogeneity in psychiatric research. *Journal of Psychiatry and Neuroscience*, 45(1):3–6, 2020. ISSN 14882434. doi: 10.1503.jpn/190198.

Abraham Nunes MD PhD MBA
Department of Psychiatry
5909 Veterans Memorial Lane
QEII Health Sciences Centre
Halifax, NS, B3H 2E2, Canada
E-Mail: nunes@dal.ca

[30] Yann LeCun, Leon Bottou, Yoshua Bengio, and P. Haffner. Gradient-based learning applied to document recognition. *Proceedings of the IEEE*, 86:2278–2324, 1998.

[31] Abraham Nunes, Thomas Trappenberg, and Martin Alda. We need an operational framework for heterogeneity in psychiatric research. *Journal of Psychiatry and Neuroscience*, 45(1):3–6, 2020. ISSN 14882434. doi: 10.1503.jpn/190198.

DALHOUSIE UNIVERSITY

Abraham Nunes MD PhD MBA
Department of Psychiatry
5909 Veterans Memorial Lane
QEII Health Sciences Centre
Halifax, NS, B3H 2E2, Canada
E-Mail: nunes@dal.ca

6 Tables

Table 1: Tabular representation of experimental results with ABIDE dataset. The effective number of typical deviation profiles (Π_1^β) for control (Normative) and autism-diagnosed (Clinical) subjects across various extreme value thresholds (first column).

	**Top** $100\%c$ **(Absolute)**		**Bottom** $100\%c$		**Top** $100\%c$	
**Threshold** c	**Normative** Π_1^β	**Clinical** Π_1^β	**Normative** Π_1^β	**Clinical** Π_1^β	**Normative** Π_1^β	**Clinical** Π_1^β
0.01	46.4 [44.4,48.4]	41.1 [38.8,43.4]	43.9 [40.8,46.0]	41.7 [38.0,44.2]	46.8 [43.6,49.4]	39.9 [37.8,42.1]
0.02	35.5 [34.0,36.8]	31.5 [30.2,32.6]	33.9 [32.1,35.1]	31.6 [30.4,32.9]	34.6 [33.2,37.0]	30.3 [29.1,31.7]
0.03	28.1 [27.3,28.9]	25.9 [25.2,26.9]	27.5 [26.2,28.3]	25.4 [24.8,26.7]	27.5 [26.2,28.2]	25.1 [24.0,26.1]
0.05	17.4 [16.9,17.7]	16.6 [16.2,17.0]	17.4 [17.1,17.7]	16.6 [16.2,16.9]	17.3 [17.0,17.7]	16.0 [15.6,16.5]
0.07	12.6 [12.5,12.8]	12.1 [11.9,12.3]	12.7 [12.4,12.8]	12.3 [12.1,12.5]	12.6 [12.4,12.8]	11.8 [11.6,12.1]

DALHOUSIE UNIVERSITY

Abraham Nunes MD PhD MBA
Department of Psychiatry
5909 Veterans Memorial Lane
QEII Health Sciences Centre
Halifax, NS, B3H 2E2, Canada
E-Mail: nunes@dal.ca

7 Figures

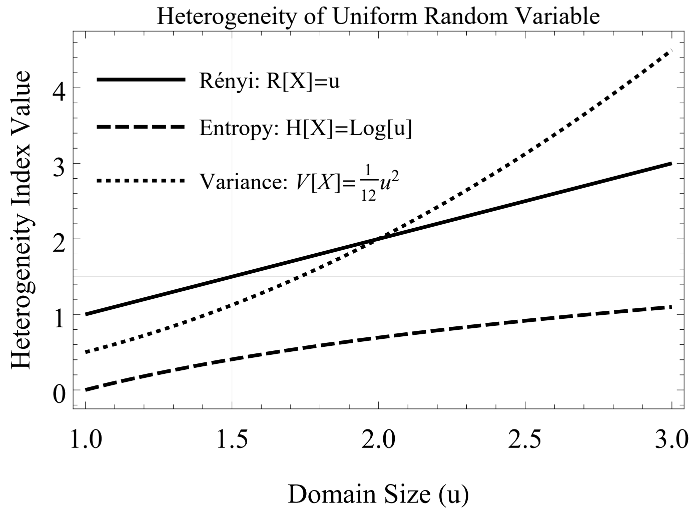

Figure 1: **Linear scaling of Rényi Heterogeneity.** Demonstration of linear scaling of Rényi heterogeneity, in comparison to the variance and entropy on a unidimensional uniform distribution with domain size u (i.e. the domain begins at the origin). Vertical and horizontal gridlines are set at 1.5 to illustrate that the Rényi heterogeneity increases by 50% when the domain size increases by 50%.
